# Athothis: A Satire on Modern Medicine

**Published:** 1888-03

**Authors:** 


*Athothis: a Satire on Modern Medicine.* By THOMAS C. MINOR. Pp. 194. Cincinnati: R. CLARKE & CO. 1887.

*Athothis* is a decidedly clever book, and has only just missed being a work of high art. The satire is exceedingly scathing, and thoroughly just; the attack might have been a little more effectual had the weapons been more sharp and more delicate. Still, it is delightful reading, and, to anyone moderately familiar with the history of medicine, is pointedly suggestive as well.

We shall not disclose the scheme and plot of the book, as this would destroy much of the pleasure of its readers. It is full of good things. The hero, Athothis, had met Saint Augustine about a hundred years previously. "He [the Saint] had transmigrated to a gnat-fly, and was busily engaged in annoying a Papal bull by discussing the Manichean heresy." "A new malady makes ravages that surprise the world." "Blessed are the poor, for they shall receive the first-fruits of medical wisdom." "The modern commercial world judges all professional men by its own standard, and considers pecuniary success the only real test of ability." "The science of modern schools of medicine, that are mere reflection son death." The pith, however, resides in paragraphs rather than in sentences, and in arguments rather than in aphorisms.

We are loth to find fault where so much is praiseworthy. The author has noted much, but we doubt if he has studied deeply the lore of the ancients. Many little slips suggest rather than prove that Routh's maxim, "Verify your references," has not been closely followed. "Pythagoris" is possibly right, instead of "Pythagoras;" "Banhinus," "Hortens," and several other words may simply be printer's errors overlooked. But "it is considered the thing in hightoned social circles" is bad taste. The sentence, "I might assert that the April showers that bring forth May flowers are merely the result of spiritual renal action, but forbear, lest I be accused of indelicacy," having been written, cannot be withdrawn; it is vulgar. There are a good many grammatical mistakes. All these defects jar on the mind which tries to look on the book as an artistic and finished production. Nevertheless, as being full of unpleasant truths, which each reader will, no doubt, apply to his neighbour with much complaisant unction, we foretell much enjoyment from its perusal.

